# Helicobacter pylori Infection as a Risk Factor for Hepatocellular Carcinoma: A Case-Control Study in Ethiopia

**DOI:** 10.1155/2018/1941728

**Published:** 2018-12-02

**Authors:** Hailemichael Desalegn Mekonnen, Henok Fisseha, Tewodros Getinet, Fisseha Tekle, Peter R. Galle

**Affiliations:** ^1^Medical Department, St. Paul's Hospital Millennium Medical College, Addis Ababa, Ethiopia; ^2^Myungsung Christian Medical Center, Addis Ababa, Ethiopia; ^3^University Medical Center Mainz, 55131 Mainz, Germany

## Abstract

*Background and Aims. *Hepatocellular carcinoma is a major cause of cancer death worldwide, accounting for over half a million deaths per year. Its incidence varies with geographic locations and the type of etiologic factors. In Ethiopia, unidentified causes of liver disease are of sizeable proportion. Recent studies have shown an association of H. pylori infection with different spectrums of chronic liver disease. This study was conducted at St. Paul's Hospital Millennium Medical College in Ethiopia and assesses liver cancer and the association with H. pylori infection.* Method. *A prospective case-control study conducted on patients with chronic liver disease presenting with a suspicious liver lesion and diagnosed to have HCC in the Gastrointestinal (GI) Clinic of St. Paul's Hospital MMC from Dec 30, 2016, to Nov 1, 2017 G.C. Descriptive surveys on clinical history and physical examination and laboratory profiles were obtained, and the clinical course of the patients including the type of treatment was followed prospectively. Control cases were taken from adult patients without evidence of liver disease in the internal medicine clinic coming for routine evaluation. After collection data were analyzed using SPSS version 23 and associations were assessed using chi-square test. Binary logistic regression was used to assess the association of HCC with different variables and H. pylori infection. All variables with p-value <0.05 were considered as statistically significant.* Results. *One hundred twenty patients were analyzed with equal representation of cases and controls. The majority of patients with HCC were male with a mean age of 36 years. Older age adjusted Odds Ratio (AOR) (95%CI, p-value) 1.07(1.03-1.09, <0.001), viral hepatitis B (AOR) (95%CI, p-value) 6.19 (1.92-19.93, 0.002), and H. pylori infection (AOR) (95%CI, p-value) 5.22 (2.04–13.31, <0.001) were statistically significantly associated with HCC.* Conclusion. *H. pylori infection is associated with HCC in this case-control study. This study supports the emerging evidence of H. pylori association with other extra-gastric manifestations.

## 1. Introduction

Liver cancer is the fifth most common cancer in men and the seventh in women. Most of the burden of disease (85%) is borne in developing countries, with the highest incidence rates reported in regions where infection with hepatitis B virus (HBV) is endemic: Southeast Asia and sub-Saharan Africa, and it is associated with a median survival of less than one year [1]. In addition to HBV infection, major risk factors for hepatocellular carcinoma include infection with hepatitis C virus (HCV), alcoholic liver disease, and nonalcoholic fatty liver disease [2].

H. pylori is the most common infectious agent worldwide and is probably one of the most ancient bacteria known to affect humans. It has been identified as the major causative agent for gastroduodenal pathologies [3]. In recent years, a possible role of H. pylori infection for several extra-gastric effects has been discussed including neurodegenerative, metabolic, and cardiovascular conditions, as well as hepatobiliary, pancreatic, and colorectal diseases [4–7].

Experimental studies in mice have shown that different species of Helicobacter has been extracted from bile, liver tissues, gall bladder, and mice with persistent hepatitis supporting likely colonization and potential impact in these organs [8–10].

When we see the burden, data from developing countries is incomplete, as patients are not properly registered and present late. From US data, estimated new case of HCC in 2018 is 42, 200, which is 2.4% of all new cancer cases. The estimated death in US, where advanced management is available, is estimated to be 30, 200, which is 5% of all cancer deaths. The percent surviving 5 years is estimated to be 17.7% [11].

European nested case-control study to assess risk factors for HCC has identified smoking to be more significantly associated with HCC, presenting in nearly half of the patients, than chronic HBV and HCV infections [12].

The burden of primary liver cancer in Ethiopia has been assessed more than four decades ago in a study on 38 cases by postmortem examination; a strong association with postnecrotic cirrhosis suggested a hepatotoxic agent as causal factor. During the observational period of three years this study demonstrated primary liver cancer to account for 20 % of the total mortality. The majority of the patients (79 %) were in the age of 41 to 60 years. [13]

Another Ethiopian Hospital based analysis around 15 years ago defined acute viral hepatitis, chronic hepatitis, liver cirrhosis, and hepatocellular carcinoma as underlying disease entities in 12% of the admissions on medical wards and as a leading causes of mortality (31%) [14, 15].

In another retrospective study in one of the major teaching hospitals in Ethiopia, evaluating 51 cases of HCC, HBV, and HCV were the most prevalent causes and most patients (88 %) presented with symptoms in an advanced stage of the disease. In this study, a sizeable fraction of patients do not have the conventional risk factors of liver disease [16].

An African consortium study to identify major risk factors and survival for hepatocellular carcinoma showed liver cancer in almost all African countries except Egypt to be associated with dismal prognosis and rapid progression [17]. Additional confounding factors in addition to the known conventional risk factors have been recommended. H. pylori is considered as a class I carcinogen for gastric cancer by the World Health Organization (WHO). The bacterium is estimated to infect nearly half of the world's population. The infection rate in Ethiopia is even higher with nearly 87 % of seropositive individuals at the age of 12 years and around 90 % of adults. The infection is mostly asymptomatic and individuals do not seek treatment until complications develop [18].

A possible contribution of Helicobacter spp. in the pathogenesis of chronic liver diseases (CLD) and liver carcinoma has been discussed after the isolation of this bacterium from liver samples of a variety of mammals [8–10]. Furthermore, different studies have described an association of H. pylori infection with liver diseases, including hepatocellular carcinoma (HCC) and an association between H. pylori infection and mortality from hepatocellular cancer has been observed in China [19].

Additional evidence comes from a very high prevalence of antibodies against H. pylori as well as the presence of this bacterium in patients with cirrhosis, which further supports a contribution of H. pylori to hepatocarcinogenesis [20–23]. Finally, H. pylori have been detected in liver tissue samples of HCC patients [24, 25]. To our knowledge the association between H. pylori and HCC has not been studied in Africa.

In this study, we investigated a possible correlation between H. pylori infection and HCC in one of the major teaching hospitals in Ethiopia.

## 2. Methods and Materials

### 2.1. Study Area and Period

The study was conducted in St. Paul's Hospital Millennium Medical College (SPHMMC), Gastrointestinal Unit, Addis Ababa, Ethiopia, from Dec 30, 2016, to Nov 1, 2017.

### 2.2. Study Population

The study included all consenting patients presenting to St. Paul's Hospital Millennium Medical College (SPHMMC) Gastrointestinal Unit and satisfying the study inclusion criteria. The population of patients was classified into cases (patients with HCC) and controls (absence of HCC, no clinical evidence of liver disease). After ethical clearance was obtained consenting, cooperative adults were included in the analysis.

#### 2.2.1. Inclusion Criteria

- Age above 18 Years
- Confirmed HCC (Cases)
- Absence of HCC and no clinical evidence of liver disease (Controls)

#### 2.2.2. Exclusion Criteria

- H. pylori stool Ag test in those with recent antibiotic or PPI exposure in the last 2 weeks
- Patients with extra hepatic malignancies
- Critically ill patients with presumed life expectancy below 4 weeks

### 2.3. Study Design

A prospective case-control study was employed.

### 2.4. Sample Size Determination and Sampling

The sample size was determined using the Fleiss 1981 formula with continuity correction and calculated using Epi InfoTM software version 7. The required database including proportions of controls exposed to H. pylori (P0= 10%) and proportions of cases exposed to H. pylori (P1= 45%) was taken from previous study [25]. The final sample size was defined with 95% confidence and 80% power, a 1:1 case to control ratio, and 10 % contingency and was calculated to be 66 (33 cases and 33 controls). By considering the rule of thumb for the number of variables and corresponding sample size, the final sample size considered for this study was 120 (60 cases versus 60 controls) and there were 5 variables considered for the regression analysis.

For sample selection the study population was divided into cases and controls, including 60 adult patients coming to the internal medicine outpatient department with no clinical liver disease as controls and 60 patients with confirmed HCC. Samples were selected prospectively from consecutive patients. Controls were individuals without hepatocellular carcinoma on routine analysis including clinical examination, abdominal ultrasound and normal liver functions. All patients were evaluated by clinical examination and laboratory tests for the presence of liver disease including viral markers and liver function tests and risk factor assessment (predesigned questionnaire) and samples were additionally tested for H. pylori infection. In detail, complete blood count (Celldyn-1800 and Symex KX-2), blood chemistry (Cobas-Integra 400 plus (Roche)), alpha-fetoprotein (Cobas e411), H. pylori stool antigen (Novatest (Cassette)), HBsAg (Determine), and HCV-Ab (Healegen) were determined.

### 2.5. Data Analysis

The collected data were entered into and analyzed by SPSS version 23. Descriptive statistics were performed to assess frequencies and percentages of investigated variables in the study. Multiple binary logistic regression that takes into account the binary nature of the outcome of interest and that can control confounding factors was used to identify factors associated with HCC. AORs were used to quantify the magnitudes of association and all variables with a p-value of < 0.05 are considered as statistically significant associated factors.

## 3. Results

### 3.1. Patient Characteristics

Among the enrolled 120 patients, 60 were cases with HCC. Among these, 34 (56.7%) were male and 48 (80%) older than 35 years. Concerning the ethnicity, the largest subgroups were Amhara 25 (44%) followed by Oromo 20 (35%). One-third had higher-level educational status or worked as government employee, 40 (69%) were married, and the majority denied alcohol consumption and use of herbal forms of therapy (see Table 1).

### 3.2. Comparison of Variables between Cases and Controls

The mean age was 51 years for controls and 36 years for cases of HCC. The gender distribution was comparable with 32 (53%) and 34 (56%) males for controls and cases, respectively. Nine (15 %) of controls were tested positive for HBsAg without clinical signs of liver disease and normal laboratory tests including transaminases. The percentage of HBsAg positive patients in the HCC case group was higher (20 patients (33.3%)). One patient in the control groups (1.7%) was positive for HCV, compared to 16 (26.7%) HCV-Ab positive patients in the HCC group. A positive stool test for H. pylori infection was obtained in 14 (23.3%) of the controls compared to 37 (61.7%) in the cases diagnosed with HCC. (Table 2)

### 3.3. Factors Associated with HCC

On a multiple binary logistic regression model, age (per year increasement) (AOR) (95%CI, p-value) 1.07(1.03-1.09, <0.001), viral hepatitis of HBsAg (AOR) (95%CI, p-value) 6.19(1.92-19.93, 0.002), and H. pylori infection (AOR) (95%CI, p-value) 5.22(2.04–13.31, <0.001) were statistically significantly associated with HCC.

## 4. Discussion

Hepatocellular carcinoma is one of the most common malignancies in the world. It is associated with high mortality. In different African countries it is responsible for early mortality and the understanding of contributing risk factors is vital. In this study we have investigated H. pylori infection as a possible associated factor in addition to the conventional causes of the disease. This study confirms that HCC occurred more frequently in male patients and affects younger age groups compared to the control groups. As the main result, in our study, H. pylori infection is more prevalent in HCC patients than in controls and, thus, appears as an associated factor for HCC in addition to HBV and HCV infection.

In a retrospective study from Ethiopia aiming to identify major associated causes for HCC, nearly half of the patients had no known underlying etiology and most of the patients present with advanced stage of the disease [16]. In our study 33 % were positive for HBV and 26.7 for HCV, leaving a large proportion without a defined risk factor.

In a large African consortium study comprising 8 countries, HBV associated hepatitis was the predominant disease and associated with early age, male sex, and poor prognosis [19]. This study has also urged African studies to identify other potential synergistic factors contributing to HCC development and progression, such as aflatoxin exposure. Another potential mechanism may be an unidentified (co)infection resulting in an inflammatory environment propagating HCC. A potential agent is H. pylori. This infection rarely results in clinical symptoms and individuals do not seek treatment until tested and identified.

The mechanism of H. pylori colonization of the human liver is postulated to be due to bacterial translocation from the stomach through the portal system, especially in the advanced stages of chronic liver disease when portal hypertension develops [26, 27]. Furthermore, bacteria can reach the liver via circulating phagocytes and macrophages or retrograde transfer from the duodenum [28]. This is further supported by reports on positive H. pylori cultures from liver samples of patients with HCC, supporting the concept of hepatic colonization [29]. A specific role of H. pylori in this context is further supported by the failure to identify other bacteria in the digestive tract associated with human liver carcinogenesis [30, 31].

H. pylori as an associated factor of chronic liver disease has been studied in different parts of the world. In China, in 67 rural areas epidemiological evidence showed this bacterium to be associated with an increased risk of death from liver cancer. Sequence analysis, comparing liver, and gastric samples demonstrated similarity, suggesting that gastric colonization with H. pylori is the cause of liver colonization. [32]

Moreover, H. pylori may play a role in the progression of HCC to advanced stage, supported by a higher prevalence in more advanced stages of HCC [33].

Other studies investigated the coinfection of HBV and H. pylori infection and showed a higher prevalence of anti-H. pylori antibodies in patients infected with HBV. Furthermore, H. pylori infection seems to be associated with later stages of chronic liver disease as DNA was detected in >60% in cirrhotic liver and 90% in HCC tumor tissue compared to 4.2 % of controls and 3.5% of noncirrhotics [27]. This could be explained by a synergistic cooperation between the bacterium and other pathogens in the development of HCC.

In this study, H. pylori were identified in 23.3% of the controls and 61.7% of patients with HCC.

We recommend further studies to assess the impact of the coinfection on hepatocarcinogenesis and progression of HCC and the potential role of eradication of H. pylori in the effect of HCC.

## Figures and Tables

**Table 1:** Baseline characteristics of patients diagnosed with HCC and controls (n=60).

Characteristics	**No HCC**	**HCC**
Gender		
Male	32(53.3)	34(56.7)
Female	28(46.7)	26(43.3)

Age Groups		
18-35	37(62.7)	12(20)
36-45	7(11.9)	15(25.0)
> 45	15(25.4)	33(55.0)

Ethnicity*∗*		
Oromo	26(43.3)	20(33.3)
Amhara	19(31.6)	25(41.7)
Tigre	8(13.3)	9(15.0)
South	5(8.3)	5(8.3)
Others	2(3.5)	1(1.7)

Educational Level*∗*		
Illiterate	10(16.7)	14(25.0)
Read/write only	11(18.3)	7(12.5)
Elementary	4(6.7)	6(10.7)
Secondary/High school	8(13.3)	7(12.5)
Higher Education	21(35.0)	19(33.9)

Occupation		
House-wife	12(20.0)	20(33.3)
Government employee	22(36.7)	18(30.0)
Daily Laborer	8(13.3)	4(6.7)
Merchant/Trader	6(10.0)	6(10.0)
Farmer	8(13.3)	5(8.3)
Jobless	4(6.7)	7(11.7)

Marital Status		
Married	42(70.0)	40(66.7)
Single	10(16.7)	12(20.0)
Divorced	8(13.3)	5(8.3)
Widowed	-	3(5.0)

Alcohol History		
No	33(97)	53(88.3)
yes	1(3)	7(11.7)

Herbal Medication use		
No	60 (100)	52(86.7)
Yes		8(13.3)

*∗*: missing values-6; HCC: hepatocellular carcinoma.

**Table 2:** Odds ratios of HCC according to baseline variables (n=120).

	Diagnosis of HCC		Unadjusted		Adjusted	
	NO HCC	HCC	P-value	OR(95%CI)	P-value	OR(95% CI)
Mean Age(+/- sd)	51	36	<0.001	1.07(1.03-1.11)	<0.001	1.06(1.03-1.09)

Gender						
Male	32(53.3)	34(56.7)	0.71	0.87(0.42-1.79)	0.91	0.94(0.35-2.56)
Female	28(46.7)	26(43.3)				

HBSAg						
Non-reactive	51(85)	40(66.7)	0.022	2.83(1.16-6.89)	0.002	6.19(1.92-19.93)
Reactive	9(15)	20(33.3)				

H. pylori						
Negative	46(76.7)	23(38.3)	<0.001	5.29(2.39-11.68)	<0.001	5.22(2.04-13.31)^ *∗*^
Positive	14(23.3)	37(61.7)				

*∗*Significant at 5% level of significance.

## Data Availability

The data used to support the findings of this study are available from the corresponding author upon request.

## References

[1] El-Serag H. B., Rudolph K. L. (2007). Hepatocellular carcinoma: epidemiology and molecular carcinogenesis. *Gastroenterology*.

[2] Sherlock S. (1994). Viruses and hepatocellular carcinoma. *Gut*.

[3] Franceschi F., Gasbarrini A. (2007). Helicobacter pylori and extragastric diseases. *Best Practice & Research Clinical Gastroenterology*.

[4] Roubaud-Baudron C., Krolak-Salmon P., Quadrio I., Mégraud F., Salles N. (2012). Impact of chronic *Helicobacter pylori* infection on Alzheimer's disease: preliminary results. *Neurobiology of Aging*.

[5] Venerito M., Selgrad M., Malfertheiner P. (2013). Helicobacter pylori: Gastric Cancer and Extragastric Malignancies - Clinical aspects. *Helicobacter*.

[6] Tebbe B., Geilen C. C., Schulzke J.-D., Bojarski C., Radenhausen M., Orfanos C. E. (1996). Helicobacter pylori infection and chronic urticaria. *Journal of the American Academy of Dermatology*.

[7] Zentilin P., Seriolo B., Dulbecco P. (2002). Eradication of Helicobacter pylori may reduce disease severity in rheumatoid arthritis. *Alimentary Pharmacology & Therapeutics*.

[8] Fox J. G., Yan L. L., Dewhirst F. E. (1995). Helicobacter bilis sp. nov., a novel Helicobacter species isolated from bile, livers, and intestines of aged, inbred mice. *Journal of Clinical Microbiology*.

[9] Fox J. G., Yan L., Shames B., Campbell J., Murphy J. C., Li X. (1996). Persistent hepatitis and enterocolitis in germfree mice infected with Helicobacter hepaticus. *Infection and Immunity*.

[10] Franklin C. L., Beckwith C. S., Livingston R. S. (1996). Isolation of a novel Helicobacter species, Helicobacter cholecystus sp. nov., from the gallbladders of Syrian hamsters with cholangiofibrosis and centrilobular pancreatitis. *Journal of Clinical Microbiology*.

[11] Surveillance Epidemiology (SEER) stat database: incidence – SEER 17 regs limited-use, Nov 2006 sub (1973–2004 varying). https://www.SEER.cancer.gov.

[12] Journal of the National Cancer Institute (2011). Hepatocellular Carcinoma Risk Factors and Disease Burden in a European Cohort: A Nested Case–Control Study. *Journal of the National Cancer Institute*.

[13] Pavlica D., Samuel I. (1970). Primary carcinoma of the liver in ethiopia: A study of 38 cases proved at post-mortem examination. *British Journal of Cancer*.

[14] Tsega E., Hansson B., Krawczynski K., Nordenfelt E. (1992). Acute Sporadic Viral Hepatitis in Ethiopia: Causes, Risk Factors, and Effects on Pregnancy. *Clinical Infectious Diseases*.

[15] Tsega E., Nordenfelt E., Hansson B. G. (1995). Hepatitis C virus infection and chronic liver disease in Ethiopia where hepatitis B infection is hyperendemic. *Transactions of the Royal Society of Tropical Medicine and Hygiene*.

[16] Mekonnen H. D. E., Sharma S., Shewaye A., Feld J., Lulu E. (2015). Major Risk Factors, Clinical And Laboratory Characteristics of Patients with Hepatocellular Carcinoma; A Retrospective Study at Tikur anbassa hospital, Addis Ababa university, Addis Ababa, Ethiopia. *Ethiopian Medical Journal*.

[17] Schmulson M. J., De Leon G., Kershenovich A., Vargas-Vorackova F., Kershenobich D. (1997). Helicobacter pylori infection among patients with alcoholic and nonalcoholic cirrhosis. *Helicobacter*.

[18] Siringo S., Vaira D., Menegatti M. (1997). High prevalence of Helicobacter pylori in liver cirrhosis: Relationship with clinical and endoscopic features and the risk of peptic ulcer. *Digestive Diseases and Sciences*.

[19] Ju Dong Y., Essa A., Ashraf O. (2016). Characteristics, management, and outcomes of patients with hepatocellular carcinoma in Africa: a multicountry observational study from the Africa Liver Cancer Consortium. *The Lancet*.

[20] Fan X. G., Zou Y. Y., Wu A. H. (1998). Seroprevalence of Helicobacter pylori infection in patients with hepatitis B. *British Journal of Biomedical Science*.

[21] Pellicano R., Leone N., Berrutti M. (2000). *Helicobacter pylori* seroprevalence in hepatitis C virus positive patients with cirrhosis. *Journal of Hepatology*.

[22] Avenaud P., Marais A., Monteiro L. (2000). Detection of Helicobacter species in the liver of patients with and without primary liver carcinoma. *Cancer*.

[23] Nilsson H., Mulchandani R., Tranberg K., Stenram U., Wadström T. (2001). Helicobacter species identified in liver from patients with cholangiocarcinoma and hepatocellular carcinoma. *Gastroenterology*.

[24] Dore M. P., Realdi G., Mura D., Graham D. Y., Sepulveda A. R. (2002). Helicobacter infection in patients with HCV-related chronic hepatitis, cirrhosis, and hepatocellular carcinoma. *Digestive Diseases and Sciences*.

[25] Verhoef C., Pot R. G. J., De Man R. A. (2003). Detection of identical Helicobacter DNA in the stomach and in the non-cirrhotic liver of patients with hepatocellular carcinoma. *European Journal of Gastroenterology & Hepatology*.

[26] Tu Q. V., Okoli A. S., Kovach Z., Mendz G. L. (2009). Hepatocellular carcinoma: Prevalence and molecular pathogenesis of Helicobacter spp.. *Future Microbiology*.

[27] Tsuneyama K., Harada K., Kono N. (2001). Scavenger cells with Gram-positive bacterial lipoteichoic acid infiltrate around the damaged interlobular bile ducts of primary biliary cirrhosis. *Journal of Hepatology*.

[28] Queiroz D. M., Santos A. (2001). Isolation of a Helicobacter strain from the human liver. *Gastroenterology*.

[29] Xuan S. (2006). Helicobacter infection in hepatocellular carcinoma tissue. *World Journal of Gastroenterology*.

[30] Ito K., Nakamura M., Toda G., Negishi M., Torii A., Ohno T. (2004). Potential role of Helicobacter pylori in hepatocarcinogenesis. *International Journal of Molecular Medicine*.

[31] Abu Al-Soud W., Stenram U., Ljungh Å., Tranberg K.-G., Nilsson H.-O., Wadström T. (2008). DNA of Helicobacter spp. and common gut bacteria in primary liver carcinoma. *Digestive and Liver Disease*.

[32] Wang L., Zollinger T., Zhang J. (2013). Association between Helicobacter pylori infection and liver cancer mortality in 67 rural Chinese counties. *Cancer Causes & Control*.

[33] Pellicano R., Mazzaferro V., Grigioni W. F. (2004). Helicobacter species sequences in liver samples from patients with and without hepatocellular carcinoma. *World Journal of Gastroenterology*.

